# Does vitamin C or its combination with vitamin E improve radial artery endothelium-dependent vasodilatation in patients awaiting coronary artery bypass surgery?

**DOI:** 10.5830/CVJA-2013-046

**Published:** 2013-10

**Authors:** Alper Uzun, Umit Yener, Omer Faruk Cicek, Adnan Yalcinkaya, Adem Diken, Turgut Ozkan, Mahmut Ulas, Ozlem Yener, Aysel Turkvatan

**Affiliations:** Department of Cardiovascular Surgery, Ankara Education and Research Hospital, Ankara, Turkey; Department of Cardiovascular Surgery, Turkey Yuksek Ihtisas Education and Research Hospital, Ankara, Turkey; Department of Cardiovascular Surgery, Turkey Yuksek Ihtisas Education and Research Hospital, Ankara, Turkey; Department of Cardiovascular Surgery, Turkey Yuksek Ihtisas Education and Research Hospital, Ankara, Turkey; Department of Cardiovascular Surgery, Turkey Yuksek Ihtisas Education and Research Hospital, Ankara, Turkey; Department of Cardiovascular Surgery, Turkey Yuksek Ihtisas Education and Research Hospital, Ankara, Turkey; Department of Cardiovascular Surgery, Turkey Yuksek Ihtisas Education and Research Hospital, Ankara, Turkey; Department of Radiology, Turkey Yuksek Ihtisas Education and Research Hospital, Ankara, Turkey; Department of Radiology, Turkey Yuksek Ihtisas Education and Research Hospital, Ankara, Turkey

**Keywords:** antioxidants, vitamin C, vitamin E, atherosclerosis

## Abstract

**Background:**

We evaluated the vasodilatory effects of two antioxidants, vitamins C (ascorbic acid) and E (α-tocopherol), on radial artery and endothelium-dependent responses in patients awaiting coronary artery bypass surgery.

**Methods:**

The study was performed in three groups. The first group took 2 g of vitamin C orally (*n* = 31, vitamin C group), the second group took 2 g of vitamin C with 600 mg of vitamin E orally (*n* = 31, vitamins C + E group), and the third group took no medication (*n* = 31, control group). After baseline measurements were taken of the radial artery lumen diameter, flow volume and lumen area in the non-dominant radial artery, occlusion was maintained for five minutes with a pressure cuff placed around the arm. The measurements were taken again at the time of deflating the cuff, and 60 seconds later. The measurements were repeated after medication in two of the groups and after placebo in the third group.

**Results:**

We compared values of the vitamin C group with those of the vitamins C + E group, and found that the latter were higher than those of the vitamin C group but not statistically significant. In the control group, there was no statistical difference.

**Conclusion:**

Vitamin C or its combination with vitamin E significantly enhanced endothelium-dependent vasodilatation in the radial circulation of patients with coronary artery disease. Its combination with vitamin E was superior to vitamin C administration alone for endothelial enhancement but this difference was not statistically significant. We hypothesised that vitamin C or its combination with vitamin E may be used as antioxidants for arterial graft patency in patients undergoing coronary artery surgery.

## Abstract

In patients with atherosclerosis, there is biochemical evidence to suggest increased oxidative stress, which results from an altered balance of endogenous pro- and antioxidants. Vitamin C, the main water-soluble antioxidant in human plasma, has been shown to reverse endothelial dysfunction in patients with ischaemic heart disease in the same way as vitamin E.^1^ It effectively scavenges superoxide and other reactive oxygen species, and plays an important role in regulation of the intracellular redox state through its interaction with glutathione.^2^

The Health Professionals Follow-up study demonstrated an inverse relationship between vitamin E intake and coronary artery disease (CAD) events.^3^ In this study, risk for CAD events in subjects in the highest quintile of vitamin E intake (median 419.0 IU/day) was significantly reduced by 41%, compared with the subjects in the lowest quintile of vitamin E intake (median 6.4 IU/day).^3^,^4^

Other studies suggest that a high dietary intake of flavonoids (polyphenolic antioxidants) naturally present in vegetables, fruits, tea and vitamin E is associated with a decline in CAD events.^5^ Nonetheless, in a subgroup analysis of patients who had undergone previous coronary artery bypass surgery, coronary artery lesion progression was less in subjects with a supplementary vitamin E intake of 100 IU per day or more, compared with patients with a lower intake.^6^

Therefore, we hypothesised that vitamin C and E would improve abnormal endothelium-dependent vasomotor function in patients with atherosclerosis. We tested this hypothesis by examining endothelium-dependent, flow-mediated radial artery dilatation before and two hours after oral administration of vitamin C or vitamin C plus vitamin E in two groups of patients awaiting coronary artery bypass surgery, compared with the control group.

## Methods

A total of 93 patients were randomly divided into three groups: group 1, vitamin C group, *n* = 31; group 2, vitamins C + E group, *n* = 31; group 3, control group, *n* = 31. Patients referred to our clinic were screened for enrolment, and patients with significant coronary artery disease were eligible for the study. In all patients, the presence of coronary artery disease was confirmed angiographically (at least one coronary stenosis > 70%).

All patients gave informed consent. The study was conducted in accordance with the policies and procedures of the Education and Planning Committee of our hospital.

All vasoactive medications were withheld for at least 12 hours before the study, and all long-acting vasoactive medications were withheld for at least 24 hours. Alcohol and caffeine were prohibited within 12 hours of the study. Patients who had a smoking history refrained from smoking at least 48 hours before the study to prevent any relevant effect of smoking.

Patients with unstable angina, severe cardiac failure, additional cardiac (valvular, congenital, etc) or peripheral vascular disease, renal and hepatic dysfunction, uncontrolled hypertension, or any other condition that would preclude withholding vasoactive medications, and patients taking antioxidant vitamin supplements, oestrogen replacement therapy or allopurinol were excluded from the study.

## Study protocol and radial artery image with ultrasound studies

The vasodilator response in the radial artery was determined from two-dimensional ultrasound images by an evaluator blinded to the study. The images were obtained with the use of a 7.0-MHz linear-array transducer (model SSA-770A Ultrasound System; Toshiba, Tokyo, Japan). Examination was performed with the patient resting supine for at least five minutes in a quiet setting. For each patient, radial artery images were obtained 2 and 5 cm above the radial styloid. This location was marked, and all subsequent images were obtained at the same location.

Images were recorded on a super-VHS videocassette recorder and radial artery diameters were measured from the tape with ultrasonic calipers. Measurements of radial artery diameter were taken from the anterior to the posterior interface between the media and adventitia at end-diastole, incident with the R-wave, on a continuously recorded ECG. The measurements were performed by one observer who was blinded to the details. At the moment of measurement, arterial blood pressures were checked for the safety of the study and were found normal (120/80 mmHg) in all patients.

In all patients, first, baseline Doppler ultrasound images were obtained (measurement 1: D1, FV1, A1). The radial artery of the non-dominant arm was scanned in transverse section and the lumen diameter (D1) was measured. Then, flow volume (FV1) and lumen area (A1) were measured in longitudinal section and all measurements were recorded.

Second, a blood pressure cuff, placed at the proximal portion of the arm, was inflated to occlusive pressure (200 mmHg), and occlusion was maintained for five minutes to induce hyperaemia. The cuff was then rapidly deflated and pulsed Doppler signals and measurements were recorded at the moment of cuff deflation for 15 seconds (measurement 2: D2, FV2, A2). Finally, two-dimensional and pulsed-Doppler measurements were again obtained and recorded 60 seconds after cuff deflation (measurement 3: D3, FV3, A3).

In group l, patients were then given 2 g oral vitamin C (Redoxon, Bayer, Germany, 1 000-mg tablets) and a repeat radial ultrasound study was performed two hours after oral administration to evaluate its effect on radial artery vasodilation in the first 31 patients. These measurements were accepted as study measurements for group 1 (measurements 4, 5, 6 for group 1: D4, 5, 6; FV4, 5, 6; A4, 5, 6).

In group 2, the next 31 patients were given orally 2 g vitamin C and 600 IU vitamin E (Ephynal, Roche, France, 300 IU, oral capsules), and a repeat radial ultrasound study was performed two hours after oral administration to evaluate their effects on radial artery vasodilation. These measurements were accepted as study measurements for group 2 (measurement 4, 5, 6 for group 2: D4, 5, 6; FV4, 5, 6; A4, 5, 6). In group 3, patients took no medication and the measurements were repeated.

Finally, the increasing values of D, FV and A of the two study groups were compared and the mean difference of their increasing values was measured (Table 1). In the control group, the results of the measurements were almost the same and there was no statistically significant difference between measurements (*p* > 0.05).

**Table 1:** Increasing Values Of Flow Volume, Area And Diameter

	*Vitamin C (mean value)*	*Vitamin C+E (mean value)*	p*-value*
FV4–FV1	1.322	1.677	0.542
A4–A1	8.096	7.929	0.952
D4–D1	2.225	2.548	0.651
FV5–FV2	2.322	2.354	0.968
A5–A2	9.064	13.364	0.211
D5–D2	2.225	2.741	0.454
FV6–FV3	1.322	1.871	0.378
A6–A3	6.612	10.806	0.378
D6–D3	2.225	2.387	0.790

FV1, FV4, baseline flow volume before and after drug intake, respectively; FV2, FV5, flow volume at the moment of cuff deflation before and after drug intake, respectively; FV3, FV6, flow volume 60 seconds after cuff deflation before and after drug intake, respectively. A1, A4, baseline area before and after drug intake, respectively; A2, A5, area at the moment of cuff deflation before and after drug intake, respectively; A3, A6, area 60 seconds after cuff deflation before and after drug intake, respectively. D1, D4, baseline diameter before and after drug intake, respectively; D2, D5, diameter at the moment of cuff deflation before and after drug intake, respectively; D3, D6, diameter 60 seconds after cuff deflation before and after drug intake, respectively.

## Statistical analysis

Results were analysed using SPSS software (SPSS 18.0.1 for Windows; SPSS, Chicago, IL, USA). Comparisons of groups were performed with the Student’s t-test and non-parametric Mann-Whitney *U*-test for continuous variables and the Fisher exact test for discrete variables, as appropriate. Pre–post comparisons with paired observations were analysed using the Wilcoxon signed-ranks test.

Linear regression models were built with least-squares methods using all variables and also with a backward elimination approach, starting with all variables that were significant (*p* < 0.05) in univariate relationships and eliminating variables with *p*-values greater than 0.05, in a stepwise fashion.

## Results

Ninety-three patients were included in the study from 1 February to 31 August 2008. Patient characteristics are summarised in Table 2. Median age, male/female gender, and left ventricular ejection fraction were similar in the three groups (*p* > 0.05). The presence of diabetes was similar in the study groups, but scarce in the control group. A history of smoking in the vitamin C group (group 1) was higher than in the vitamins C + E group (group 2) and the control group. Also, a history of mild hypertension in the vitamin C group was higher than in the vitamins C + E and control groups but it was not statistically significant.

**Table 2:** Summary Of The Key Characteristics Of The Subjects

	*Vitamin C group*	*Vitamin C + E group*	*Control group*
Age, median	56.32 ± 12.50	55.74 ± 13.36	56.20 ± 12.40
Ejection fraction	49.64 ± 12.57	51.90 ± 9.83	50.30 ± 10.47
Male/female	24/7	25/6	26/5
History of smoking	16	11	10
Diabetes mellitus	7	7	4
Mild hypertension	15	10	10

**Fig. 1.**
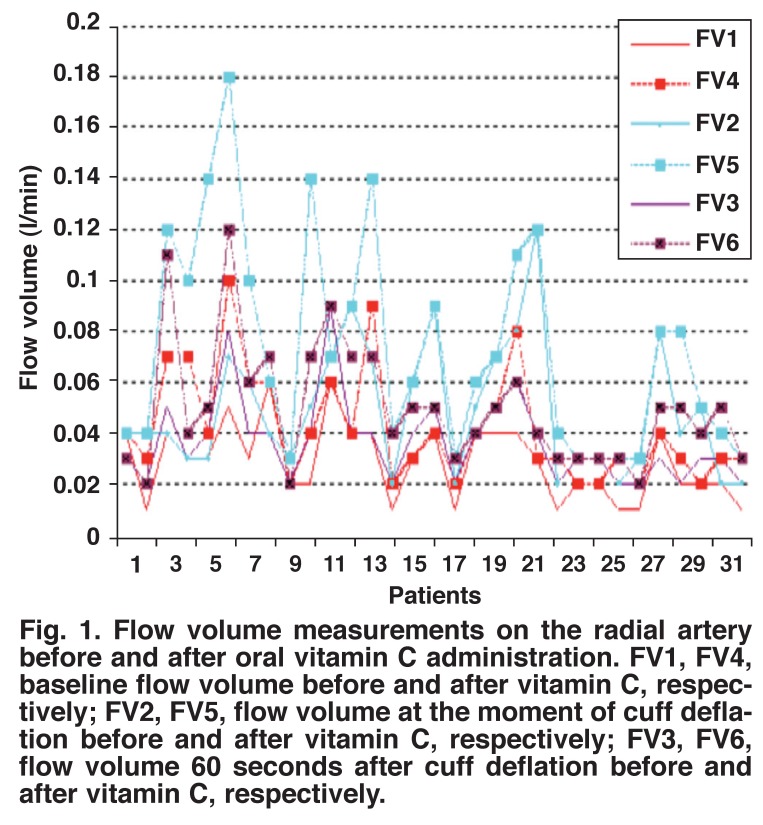
Flow volume measurements on the radial artery before and after oral vitamin C administration. FV1, FV4, baseline flow volume before and after vitamin C, respectively; FV2, FV5, flow volume at the moment of cuff deflation before and after vitamin C, respectively; FV3, FV6, flow volume 60 seconds after cuff deflation before and after vitamin C, respectively.

**Fig. 2.**
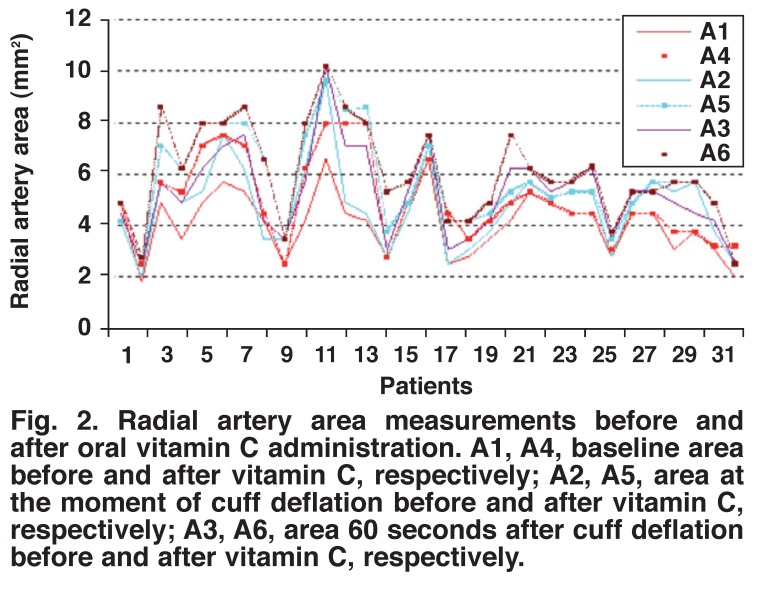
Radial artery area measurements before and after oral vitamin C administration. A1, A4, baseline area before and after vitamin C, respectively; A2, A5, area at the moment of cuff deflation before and after vitamin C, respectively; A3, A6, area 60 seconds after cuff deflation before and after vitamin C, respectively.

**Fig. 3.**
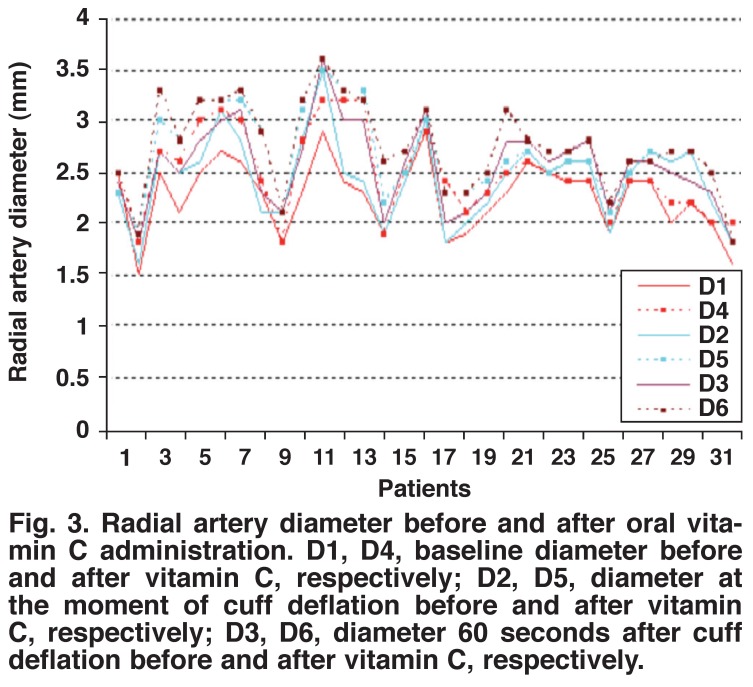
Radial artery diameter before and after oral vitamin C administration. D1, D4, baseline diameter before and after vitamin C, respectively; D2, D5, diameter at the moment of cuff deflation before and after vitamin C, respectively; D3, D6, diameter 60 seconds after cuff deflation before and after vitamin C, respectively.

**Fig. 4.**
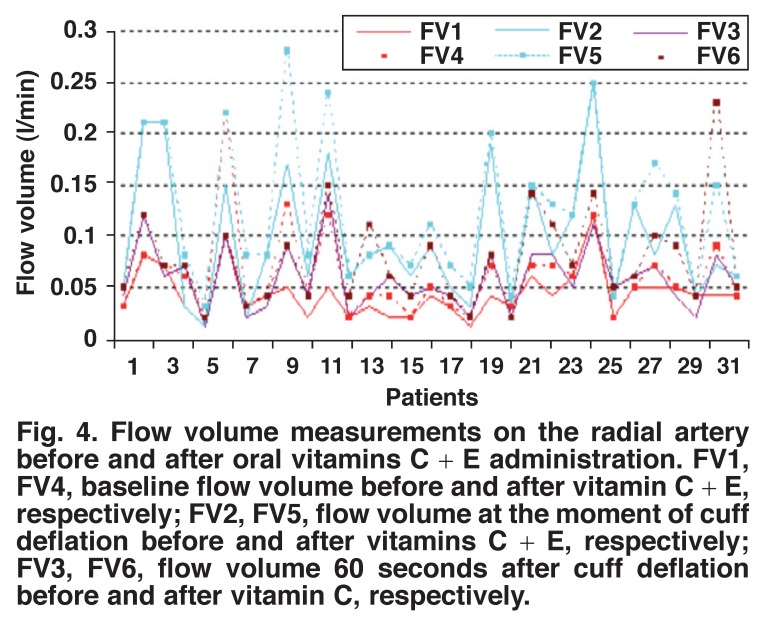
Flow volume measurements on the radial artery before and after oral vitamins C + E administration. FV1, FV4, baseline flow volume before and after vitamin C + E, respectively; FV2, FV5, flow volume at the moment of cuff deflation before and after vitamins C + E, respectively; FV3, FV6, flow volume 60 seconds after cuff deflation before and after vitamin C, respectively.

**Fig. 5.**
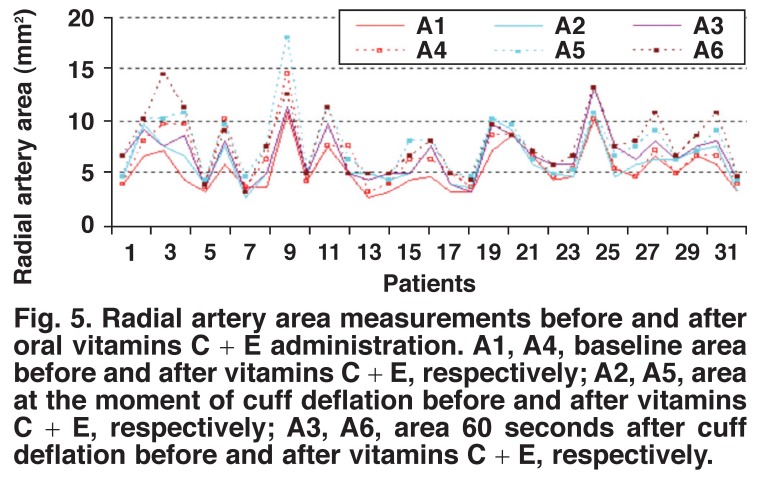
Radial artery area measurements before and after oral vitamins C + E administration. A1, A4, baseline area before and after vitamins C + E, respectively; A2, A5, area at the moment of cuff deflation before and after vitamins C + E, respectively; A3, A6, area 60 seconds after cuff deflation before and after vitamins C + E, respectively.

**Fig. 6.**
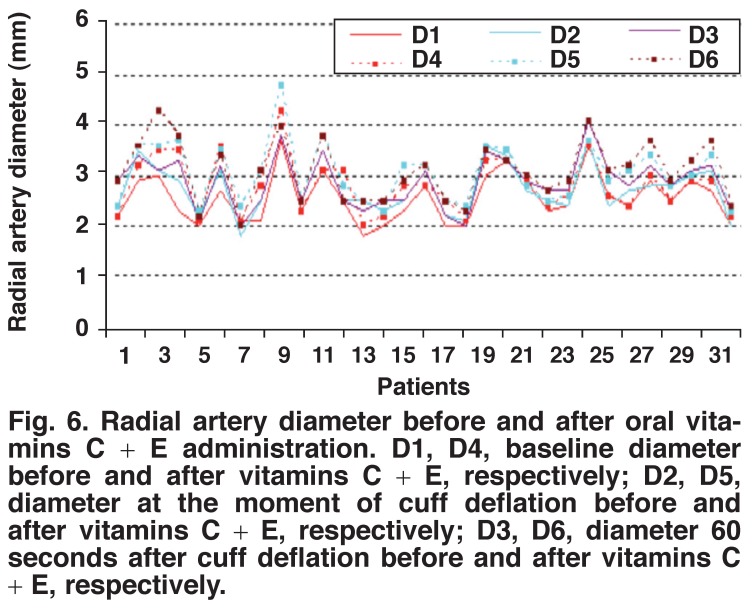
Radial artery diameter before and after oral vitamins C + E administration. D1, D4, baseline diameter before and after vitamins C + E, respectively; D2, D5, diameter at the moment of cuff deflation before and after vitamins C + E, respectively; D3, D6, diameter 60 seconds after cuff deflation before and after vitamins C + E, respectively.

Pre- and post-administration measurements of vitamin C in group 1 showed a statistically significant increase in the radial artery flow volume, lumen diameter and lumen area after two hours of vitamin C administration (Figs 1, 2, 3) (*p* < 0.001). Preand post-administration measurements of vitamins C + E in group 2 showed a statistically significant increase in the radial artery flow volume, lumen diameter and lumen area after two hours of vitamin C with vitamin E administration (Figs 4, 5, 6) (*p* < 0.001).

Patients in both the vitamin C group (measurement 4, 5, 6 for group 1) and in the vitamins C + E group (measurement 4, 5, 6 for group 2) showed statistically significant increases in the radial artery flow volume, lumen diameter and lumen area when compared with the time of measurements 1, 2 and 3. Its combination with vitamin E was superior to vitamin C administration alone for endothelium-dependent vasodilatation but this difference was not statistically significant. This is shown in Table 2.

Against the two groups, there was no statistical difference in the control group. The repeat measurements were not statistically different from the first measurements and they were also not different from the baseline measurements of the two groups.

## Discussion

Antioxidants vitamins C and E improved defective endothelial function. This has been attributed to an enhancement in the synthesis or prevention of the breakdown of nitric oxide (NO).^7^ NO plays a major role in maintaining normal tonus in the artery. This study demonstrated that administration of vitamin C or its combination with vitamin E increased endothelium-dependent dilatation in patients awaiting coronary artery bypass surgery.

The radial artery is increasingly being used as a conduit for coronary bypass grafting because of reports of long-term patency, accessibility and encouraging mid-term results.^8^-^10^ The choice of a potent vasodilator with minimal side effects appears to be an important parameter in ensuring the success of radial artery conduits. Also, imaging by Doppler ultrasound of radial artery dilatation after drug treatment is easy. Therefore we particularly used the radial artery for measurements.

A history of smoking is an important parameter that can affect the results. Its vasoconstrictor effect on the endothelium has been shown in many studies. In our study, the vitamin C group had more subjects with a history of smoking than the vitamins C + E and control groups, but this was not important because all smokers refrained from smoking for at least 48 hours before the study to prevent any significant effects of smoking.

Several large epidemiological studies have suggested that dietary intake of vitamin C is inversely associated with the risk of ischaemic heart disease.^11^ In other studies, however, vasodilation was observed to increase over a period of only two hours.^11^,^12^ We repeated the measurements after two hours of administration because Levin *et al*. showed that plasma ascorbic acid levels reached a plateau after two hours and remained elevated five hours after ingestion.^13^ On the other hand, Westhuyzen *et al*. showed that α-tocopherol concentrations after oral intake had reached the same levels by the second hour and stayed at that level for almost five hours.^14^ We therefore did not measure the blood levels of vitamins C and E.

Drossos *et al.* showed that vitamin C, like vitamin E, has a potent vasodilating effect on the radial artery.^15^ They examined the dilation of the lumen surface and colour Doppler images of the non-dominant radial artery just before and two hours after oral vitamin C administration. The results provided evidence that vitamin C was a potent vasodilator in healthy subjects, particularly in smokers. In addition, it was a superior acute vasodilating agent *in vivo* compared with diltiazem in ischaemic patients awaiting cardiac surgery.^15^

In our study, we used the same method to measure radial artery vasodilation. We took measurements at the time of cuff deflation and 60 seconds later to observe the effect of vitamin C on the endothelium.

Excessive vascular oxidative stress has been linked to impaired endothelium-dependent arterial relaxation in coronary artery disease. Keaney *et al.* showed in their study the beneficial effects of vitamin E on endothelial function.^16^

Vitamins C and E may favourably influence cardiovascular risk, but there are several important differences between these naturally occurring antioxidants. Vitamin C is water soluble, and is present in most body fluids. However, vitamin E is a lipidsoluble antioxidant.

The primary antioxidant mechanisms of these antioxidants are also distinct. The important antioxidant properties of vitamin C are its abilities to scavenge superoxide anions and to preserve reduced intracellular glutathione concentrations. Also, vitamin C is required for the regeneration of vitamin E.^17^ Vitamin C may thus prevent low-density lipoprotein (LDL) oxidation, either through the recycling of vitamin E or by scavenging free radicals directly.^18^ We therefore observed the beneficial effects of vitamins C and E on endothelial function in our study.

In a recent double-blind trial, Brown *et al.* studied simvastatin–niacin and antioxidant vitamin therapy, alone and together, for cardiovascular protection in patients with coronary disease and low plasma levels of high-density lipoprotein (HDL) cholesterol.^19^ The baseline levels of LDL cholesterol and triglycerides decreased when antioxidant vitamins were added to the simvastatin–niacin regimen. The HDL level increased by 18% in those treated with simvastatin–niacin and antioxidant vitamins. With simvastatin–niacin and antioxidant vitamin therapy, the levels of HDL2 and apolipoprotein A-I [Lp(A-I)] increased by 81%. The resistance of LDL to oxidation increased by 35%.

In an another study, Behrendt et al. showed that vitamin C and E combinations reduced cardiac transplant-associated arteriosclerosis in patients with normal or abnormal endothelial function. The magnitude of benefit was larger in patients with endothelial dysfunction.^20^

## Conclusion

This study demonstrated that oral administration of the antioxidants vitamins C and E in physiological doses may enhance endothelium-dependent vasodilatation in the radial artery of patients with coronary artery disease. However, the combination was not more effective than vitamin C alone. This finding suggests that in this setting, increased oxidative stress may be an important mechanism for impaired endothelial function. Combination of vitamins C and E may show beneficial effects on graft patency due to improved vasomotor function of the endothelium (as on radial artery or internal mammarian artery conduits) in patients undergoing coronary artery surgery.
